# New avenues of sepsis research: obtaining perspective by analyzing and comparing SSCG 2021 and J-SSCG 2020

**DOI:** 10.1186/s40560-022-00606-7

**Published:** 2022-03-07

**Authors:** Tomoaki Yatabe, Moritoki Egi, Hiroshi Ogura

**Affiliations:** 1 Department of Anesthesiology and Intensive Care Medicine, Nishichita General Hospital, 3-1-1, Nakanoike, Tokai, Aichi Japan; 2 Division of Anesthesiology, Department of Surgery Related, Kobe University Graduate School of Medicine, Kusunoki-cho 7-5-2, Chuo-ku, Kobe, Hyogo Japan; 3 Department of Traumatology and Acute Critical Medicine, Osaka University Medical School, Yamadaoka 2-15, Suita, Osaka Japan

**Keywords:** Sepsis, Septic shock, Guideline, Research

## Abstract

Recently, revisions of two sepsis guidelines, namely, the Japanese Clinical Practice Guidelines for Management of Sepsis and Septic Shock 2020 and the Surviving Sepsis Campaign Guidelines 2021, were published. Although both guidelines were created in accordance with the Grading of Recommendations, Assessment, Development and Evaluation approach, the evidence-to-decision tables differed between them. In addition, certain recommendations may differ between these guidelines for similar clinical questions because of differences in the “PICO” criteria. Other differences in recommendations between the two guidelines are due to unclear evidence, and these ambiguities may provide the basis for further sepsis research. We hope that these two guidelines will contribute to the creation of new clinical evidence in addition to supporting treatment of patients with sepsis.

Recently, revisions of two sepsis guidelines, namely, the Japanese Clinical Practice Guidelines for Management of Sepsis and Septic Shock (J-SSCG) 2020 [1, 2] and the Surviving Sepsis Campaign Guidelines (SSCG) 2021 [3, 4], were published. A new domain of “patient- and family-centered care” was added to J-SSCG 2020, while that of “long-term outcomes” was added to SSCG 2021. Thus, in addition to focusing on the acute phase of sepsis, these guidelines emphasized the importance of considering the social and long-term aspects, including post-intensive care syndrome, during its treatment.

In the process of revision, many similarities were discovered between the two guidelines. For example, both their aims were to assist in appropriate clinical decision-making to improve the prognosis of patients suffering from sepsis and septic shock. In addition, both guideline working groups included patients for better reflection of their perspectives in the guidelines. However, there are certain differences (Tables 1, 2). The target audience for both guidelines included medical professionals, such as clinicians, nurses, and pharmacists, while that for SSCG 2021 also included policymakers. In addition, SSCG 2021 considered low- and middle-income settings. For example, J-SSCG 2020 recommended that continuous renal replacement therapy (CRRT) be used for the management of hemodynamically unstable patients with sepsis [1, 2]. However, SSCG 2021 did not include a clinical question about CRRT in this population. SSCG 2021 noted that the specialized equipment, expertise, and personnel required for such continuous modalities may not be available in low- and middle-income economies [3, 4]. Therefore, SSCG 2021 might not recommend its use.

**Table 1:** Differences between the two guidelines

	J-SSCG2020	SSCG2021
Target audience	Medical professionals	Medical professionals and policymakers
Evidence to decision table	8 Domains	12 Domains
Categories of clinical question	BQ, GPS, GRADE, EC	BPS, GRADE, EC
Number of recommendations	125	93
Definition and diagnosis of sepsis	2	Appendix
Diagnosis of infection	5	1
Source control	9	2
Antimicrobial therapy	11	18
Intravenous immunoglobulin therapy	3	1
Initial resuscitation/inotropes	15	20
Corticosteroid therapy	3	1
Blood transfusion therapy	4	1
Respiratory management	6	12
Management of pain, agitation, and delirium	6	0
Acute kidney injury/blood purification	7	4
Nutrition support therapy	10	2
Blood glucose management	2	1
Body temperature control	2	0
Diagnosis and treatment of DIC	6	0
Venous thromboembolism countermeasures	3	3
ICU-AW and early rehabilitation	3	0
Pediatric considerations	13	0
Neuro intensive care	1	0
Patients-and family-centered care	7	0
Sepsis treatment system	5	4
Stress ulcer prophylaxis	2	1
Bicarbonate therapy	0	2
Long-term outcomes and goals of care	0	20

*J-SSCG* Japanese Clinical Practice Guidelines for Management of Sepsis and Septic Shock, *SSCG* Surviving Sepsis Campaign Guideline, *BQ* background question, *GPS* good practice statement, *GRADE* Grading of Recommendations, Assessment, Development and Evaluation, *EC* expert consensus, *BPS* best practice statement, *DIC* disseminated intravascular coagulation, *ICU-AW* intensive care unit-acquired weakness

**Table 2:** Differences of clinical question or recommendation between J-SSCG2020 and SSCG2021

J-SSCG2020	SSCG2021
qSOFA score	
Introduce as one of the screening tools	Recommend against using qSOFA as a single screening tool
Adrenaline in patients with sepsis/septic shock	
Suggest against using adrenaline as a second-line vasopressor	Suggest adding adrenaline as the third line agent of vasopressor
Guiding resuscitation	
Suggest using lactate levels as an indicator for initial resuscitation	Suggest guiding resuscitation to decrease serum lactate and capillary refilling time
Renal replacement therapy for hemodynamically unstable patients	
Continuous RRT should be used	No recommendation
Initiation of enteral nutrition	
Suggest initiating at an early period of acute phase (within 24–48 h)	Suggest early (within 72 h) initiation
Vitamin C in septic patients	
Suggest providing vitamin C	Suggest against using IV vitamin C
Mechanical venous thromboembolism prophylaxis	
Suggest using mechanical prophylaxis	Suggest against using mechanical VTE prophylaxis in addition to pharmacological prophylaxis

*J-SSCG* Japanese Clinical Practice Guidelines for Management of Sepsis and Septic Shock, *SSCG* Surviving Sepsis Campaign Guideline, *qSOFA* quick Sequential Organ Failure Assessment

Although both guidelines were created in accordance with the Grading of Recommendations, Assessment, Development and Evaluation (GRADE) approach, J-SSCG 2020 adopted eight domains in the evidence-to-decision (EtD) table, while in SSCG 2021, the resources required, certainty of evidence of required resources, cost-effectiveness, and equity were used for making a recommendation. Guideline recommendations were not based solely on the certainty of evidence, and both guidelines included certain recommendations in areas where no randomized controlled trials (RCTs) had been conducted. In the J-SSCG 2020, expert consensus was made based on EtD table included opinions of panel members and evidence except RCTs. On the other hand, in SSCG 2021, “in our practice statement” were made based on the majority opinion of the guideline panel.

In addition, certain recommendations may differ between these guidelines for similar clinical questions because of differences in the “PICO” criteria. The guidelines may have included studies based on different criteria for patients, problem, and population (the population that received an intervention); interventions; comparisons, controls, and comparators (interventions to compare with “I”); and outcomes (events that may occur as a result of the intervention). The resultant differences in included studies might have affected the final recommendations. For example, J-SSCG 2020 recommends enteral nutrition within 24–48 h of initiation of therapy in patients with sepsis, while SSCG 2021 recommends initiation of enteral nutrition within 72 h. As similar RCTs were included in the meta-analyses of the two guidelines, we believe that the differences in recommendations were determined by whether the intervention (the “I” in “PICO”) was set to within 72 or 48 h. Further studies on the optimal timing of the initiation and increase in nutrition and the appropriate dose are required. Similarly, the J-SSCG 2020 recommends the use of lactate as an indicator of tissue hypoperfusion during initial resuscitation, while the SSCG 2021 recommends the use of a decrease in lactate as such an indicator. This difference may also be due to differences in the PICO criteria. The J-SSCG 2020 recommendation is based on the results of systematic reviews in which lactate, or the change in serum lactate concentration was measured in the intervention group, while it seems that SSCG 2021 included studies in which the decrease in lactate was assessed as the intervention.

Although J-SSCG2020 did not include any recommendation on capillary refill time (CRT), the SSCG 2021 weakly recommends the use of CRT to guide resuscitation as an adjunct to other measures of perfusion in the absence of advanced hemodynamic monitoring [3, 4]. Previous expert consensus recommends using CRT as peripheral perfusion assessment during fluid resuscitation based on just two observational studies [5]. In addition, the ANDROMEDA-SHOCK study [6] did not show a clear effect of the measurement of CRT on mortality. Therefore, there is little clinical evidence for the recommendation of CRT in SSCG2021. Nonetheless, the SSCG 2021 prefers to recommend the use of CRT, apparently because of its physiologic plausibility, ease of measurement, non-invasive nature, and availability at no cost [3, 4]. Thus, there is a need for studies in which the effectiveness of the measurement of CRT, lactate, and decrease in lactate is compared in terms of patient-centered outcomes and cost-effectiveness in settings where lactate is easily measured.

A number of recommendations that differ between the two guidelines are based on unclear evidence, and such ambiguities may provide a basis for further sepsis research. For example, these guidelines provide different recommendations for initial resuscitation, which is a key point in sepsis treatment. The recommended first- and second-line vasopressors in both guidelines are noradrenaline and vasopressin, respectively. However, J-SSCG 2020 provided no direct comparison between the use of noradrenaline and vasopressin as first-line agents. Although no details were provided, such a comparison was made in SSCG 2021. The SSCG 2021 working group noted that there was evidence to suggest that vasopressin may be superior to noradrenaline in terms of clinical outcomes [3, 4] but recommended noradrenaline as first-line treatment after considering the higher costs and lower availability of vasopressin. Second, J-SSCG 2020 does not recommend the use of adrenaline or dopamine in adult patients with septic shock without cardiac dysfunction, while SSCG 2021 suggests the addition of adrenaline as a third-line agent after noradrenaline and vasopressin. Evidence for catecholamine selection in septic shock was insufficient for a strong recommendation in both guidelines. Therefore, further research is required to determine which catecholamines should be used in different situations.

The recommendations for vitamin C administration differ between the guidelines despite selecting many of the same sources of evidence. In both guidelines, meta-analyses indicated that the desirable effects of vitamin C outweigh the undesirable effects. However, SSCG 2021 recommended against using vitamin C as the balance of effects did not favor either vitamin C or the placebo. This decision was based on the fact that the point estimate of 90-day mortality favored the control group in the largest RCT [7]. Thus, although the results of the meta-analyses used by the two working groups were similar, they made opposing recommendations because of the difference in their final judgment of the balance of effects. The doses and durations of administration of vitamin C varied in each study. A recent meta-analysis, published after the guidelines were prepared, revealed that the duration of administration might influence mortality [8]. In addition, it was used in combination with hydrocortisone in certain RCTs. Another meta-analysis reported that combination treatment with vitamin C, hydrocortisone, and vitamin B1 was not superior to standard care or placebo in terms of mortality and a renal composite outcome [9]. Thus, further research about the effect of vitamin C on mortality in patients with sepsis is required.

New research questions may be identified when, in addition to reading the recommendations, the evidence for their rationale and the process of decision-making are analyzed. We hope that these two guidelines will contribute to the creation of new clinical evidence in addition to supporting the treatment of patients with sepsis.

## Data Availability

Not applicable.

## Abbreviations

J-SSCG: Japanese Clinical Practice Guidelines for Management of Sepsis and Septic Shock
SSCG: Surviving Sepsis Campaign Guidelines
CRRT: Continuous renal replacement therapy
GRADE: Grading of Recommendations, Assessment, Development and Evaluation
EtD: Evidence-to-decision
RCT: Randomized controlled trial
CRT: Capillary refill time
